# Cliff-dwelling plants: rare and precious species in harsh habitats

**DOI:** 10.3389/fpls.2025.1535385

**Published:** 2025-05-05

**Authors:** Zhengnan Zhao, Hongyan Sun, Si Liu, Xiao Chen, Shiwei Zhao, Jiangang Zhu

**Affiliations:** Beijing Key Laboratory of Greening Plants Breeding, Beijing Academy of Forestry and Landscape Architecture, Beijing, China

**Keywords:** cliff-dwelling plants, cliff habitats, survival mechanism, observation methods, plant conservation

## Abstract

Cliffs represent unique ecosystems that harbor diverse rare cliff-dwelling plants (CDPs), which play critical roles in enhancing biodiversity and maintaining ecological stability. Despite substantial advances in CDP research enabled by modern biological technologies, particularly in diversity assessment, taxonomic classification, and conservation strategies, the field lacks comprehensive syntheses of recent progress, impeding efficient identification of emerging research directions. This review systematically examines five key research domains: (1) The ecological and potential application value of CDPs; (2) cliff habitat ecology, focusing on abiotic factors such as microclimate, edaphic properties, and water conditions, as well as biotic components; (3) adaptive strategies of CDPs, encompassing morphological specializations, physiological resilience mechanisms, early growth stage, reproductive characteristics, molecular-level evolutionary adaptations, and genetic diversity patterns; (4) technological advancements in cliff research methodologies; and (5) CDP conservation strategies by conducting a comparative analysis between *in situ* protection measures and *ex situ* preservation approaches. Our review reveals three critical research gaps demanding immediate attention: the need for long-term ecological monitoring of cliff habitats and their microenvironmental dynamics, the imperative to understand CDPs’ physiological adaptations to extreme environments, and the necessity to elucidate molecular mechanisms governing plant-environment interactions within these unique ecosystems. Future research should prioritize the development of innovative tools, advanced technologies, and interdisciplinary methodologies to fill in existing knowledge gaps in the field. Such comprehensive efforts will not only elucidate the complex adaptation mechanisms of CDPs but also provide a scientific foundation for their effective conservation and ecosystem management.

## 1 Introduction

Cliff habitats represent one of Earth’s most distinctive and ecologically remarkable ecosystems. A significant number of rare and endemic plant species depend on cliffs for their survival (Vogler and Christoph, 2011; Jung et al., 2019), and these plants play a crucial role in enhancing regional genetic diversity and ecological value (Strumia et al., 2020). Compared to many other habitats, the soil conditions on cliffs are severe, with limited water and nutrient availability. As a result, CDPs face substantial selective pressures (Panitsa et al., 2021). On the other hand, CDPs experience less interspecific competition and are less likely to be grazed by herbivores (Valli et al., 2021). Studying CDPs requires a clear understanding of several key aspects. First, it is important to examine how the harsh conditions of cliff habitats affect plant survival. Additionally, exploring how these plants have evolved and adapted to such challenging environments over time is crucial. This paper systematically reviews five key aspects of CDP research: (1) The ecological and potential application value of CDPs; (2) the critical edaphic factors in cliff ecosystems, including soil nutrient dynamics, fungal microbiome, and hydrological conditions that affect the survival of plants; (3) the adaptive characteristics of CDPs, which encompasses their morphological specializations, physiological adaptations, growth and reproductive strategies, molecular mechanisms, and genetic diversity patterns; (4) the innovative methodologies and specialized equipment utilized in studies of CDPs; and (5) the conservation status and protection strategies for CDPs, focusing on both *in situ* and *ex situ* approaches. Through a comprehensive synthesis of advancements in CDP research, this review aims to offer critical insights and guide future investigations into the ecology, adaptation, and conservation of CDPs.

## 2 Ecological and potential application value of CDPs

Cliffs are often regarded as “last-known” ecological habitats (De Micco and Aronne, 2012), characterized by unique soil compositions, moisture regimes, and microclimatic conditions that support a high prevalence of endemic and rare plant species, thereby significantly contributing to biodiversity (Vogler and Christoph, 2011; Jung et al., 2019). These distinctive features confer exceptional ecological value to cliff ecosystems. Notably, insects play a pivotal role in the pollination and seed dispersal of many CDPs (Wu et al., 2022; García et al., 2012), underscoring their importance in maintaining ecological interactions. Concurrently, rhizosphere microorganisms facilitate the decomposition of organic matter, recycling nutrients into the soil to sustain CDP growth (Franz-Sebastian and March-Salas, 2022). In return, CDPs provide sustenance for insects and supply essential water and carbon (C) resources for microorganisms. These intricate relationships among plants, insects, fungi, and their environment collectively enhance ecosystem stability. Additionally, the extensive root networks of certain CDPs stabilize cliff faces, mitigating rockfall and erosion (Liu et al., 2007), thereby contributing to soil and water conservation. Many CDPs also exhibit remarkable adaptations, such as drought resistance (Chen et al., 2020) and salt tolerance (Lema et al., 2019), making them ideal candidates for ecological restoration and habitat reconstruction. Beyond their ecological roles, CDPs hold significant potential for pharmaceutical applications. For instance, *Adiantum nelumboides*, a species utilized in Traditional Chinese Medicine, contains bioactive compounds known to enhance immune function (Liang et al., 2023). These dual roles-ecological and medicinal-highlight the multifaceted importance of CDPs in environmental conservation and human health. Given their ecological significance, unique habitat requirements, and potential values, a fundamental question arises: Do these extreme habitats affect their essential survival needs and how do CDPs adapt to and thrive in the harsh conditions of cliff environments?

## 3 The harsh conditions of cliff habitats affect plant survival

**Figure 1:**
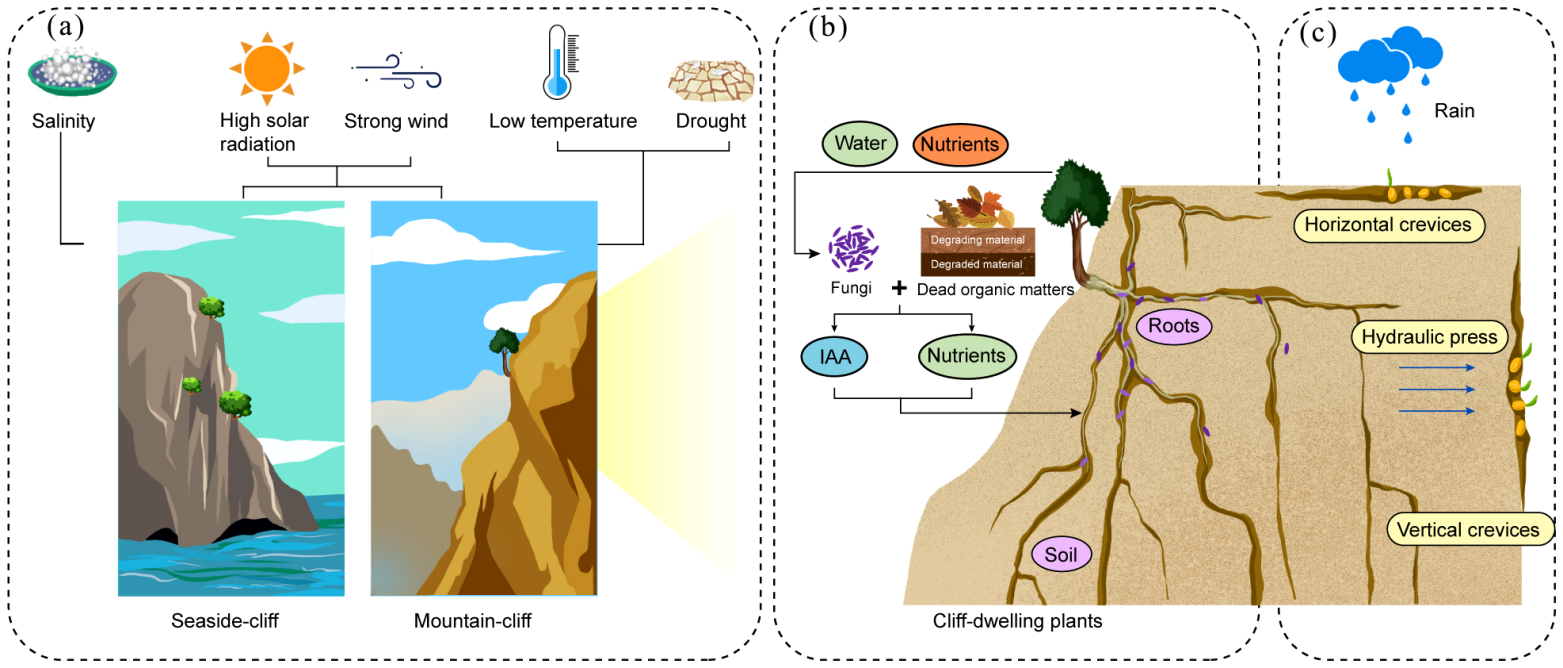
Growth environments of the CDPs. **(a)** Coastal and mountain CDPs are subjected to strong winds and high-intensity solar radiation. Coastal CDPs also face salt stress, while mountain CDPs endure drought and low-temperature stress. **(b)** The microhabitat where the CDPs thrive consists of the narrow crevices of the cliff where there is little soil, which is highly fertile. Fungi (purple-colored) within the root systems of CDPs play a crucial role in the acquisition of nutrients. **(c)** While drought is the primary environmental stress in cliff habitats, the availability of water within the cliff crevices varies depending on their orientation. Horizontal crevices are moist with high rates of seed retention. However, the seedling survival rates are low. In contrast, vertical crevices maintain relatively stable amounts of moisture owing to the water pressure, which results in a low rate retention rate of seeds but a higher seedling survival rate.

Cliff environments are extremely severe and subject the plants to intense environmental pressures. Coastal and mountainous CDPs face challenges, such as high solar radiation and strong winds (De Micco and Aronne, 2012; Pérez-Diz et al., 2023). The coastal CDPs are particularly vulnerable to salt stress (Li et al., 2023), while those on mountain cliffs are impacted by drought and low temperatures (Li W. G. et al., 2024) (**Figure 1a**). These extreme conditions have profound effects on various aspects of CDPs, including their survival, morphology, reproduction, and levels of population genetic diversity and differentiation. Despite their seemingly inhospitable nature, the cliffs can still provide the necessary conditions to support the survival of CDPs.

### 3.1 The soil conditions in the habitats affect the survival of CDPs

Rocks constitute the foundational material substrate for soil formation and development, playing a pivotal role in shaping soil types and determining their physicochemical properties (Hu and Gu, 2022). This divergence in soil properties is reflected in the distribution of CDPs: *Dionysia involucrata* thrives on granite rock cliffs (Nowak et al., 2014), while *Clematis acerifolia* is predominantly found on limestone cliffs (Zhao et al., 2023a). Although the distribution of CDPs is shaped by a multitude of factors, including temperature, climate, altitude, microenvironments, and microbial communities, soil type emerges as a critical and overarching determinant. For example, plants growing in limestone soils must contend with neutral to alkaline conditions, high concentrations of calcium, magnesium, and carbonates, limited availability of phosphorus, iron, and zinc, and frequent drought stress (Flores-Galván et al., 2024). In contrast, granite soils are typically acidic, rich in potassium and silicon, but deficient in organic matter and phosphorus, creating drier and less fertile conditions for plant growth (Hu and Gu, 2022).

### 3.2 Fungi play an important role in the growth of CDPs

Although extreme environments have traditionally been considered inhospitable to fungal survival (Gostinčar et al., 2022), growing evidence demonstrates that fungi not only persist but thrive in such conditions, including extremely cold habitats (Gunde-Cimerman et al., 2003) and arid deserts (Santiago et al., 2018). This raises an intriguing question: do cliffs, as another type of extreme environment, also harbor diverse and functionally significant fungal communities? In 2022, environmental DNA metabarcoding (eDNA) was used to examine the fungal diversity on three cliffs in Spain, revealing the presence of a remarkably diverse fungal community in these habitats. Notably, most of the fungi identified were saprotrophs, which play a crucial role in decomposing dead plant material and recycling nutrients back into the cliff crevices, thereby facilitating plant growth and sustaining the cliff ecosystem (Franz-Sebastian and March-Salas, 2022). In turn, host plants provide C sources that may enhance the survival of fungi. A study that compared two types of cliff soils- those with and without CDPs- found no significant differences in fungal amplicon sequence variant richness or composition between the two types of soil. The similarity in fungal communities may be owing to several factors. First, saprotrophic fungi in cliff habitats may prefer organic matrix components over plant material (Algora Gallardo et al., 2021). Secondly, the harsh selection pressures of the cliff environment may have a stronger influence on the fungal communities than the presence of host plants.

A series of studies have explored *Festuca rubra* subsp. *pruinosa*, a perennial plant that grows on sea cliffs, and its fungal endophytic microbiome. The microbial composition of the rhizosphere was analyzed, and it revealed a total of 135 fungal species, with *Diaporthe* and *Fusarium* identified as core components of the microbiome (Pereira et al., 2019). It has been reported that these two fungal species promote plant growth and development. The mechanisms by which *Diaporthe atlantica* supports plant growth could include the breakdown of dead roots and other organic matter into N sources and simple carbohydrates, which help the host plants to access nutrients, as well as the production of indole-3-acetic acid (IAA), which stimulates root growth (Toghueo et al., 2023) (**Figure 1b**), while *Fusarium oxysporum* can promote the growth of *Festuca rubra* subsp. *pruinosa* leaves and roots, as well as significantly reducing the content of Na^+^ in its leaves, which may be related to the mechanisms of salt tolerance in the plants (Pereira et al., 2021).

### 3.3 The direction of crevices in cliff habitats affects the survival of plants

The availability of cliff water is primarily determined by rainfall patterns and the porosity and permeability characteristics of rock formations (Aronne et al., 2018). Furthermore, the direction of cliff crevices significantly influences surface water distribution, which subsequently affects seed germination and seedling establishment during the initial developmental stages of CDPs. An 18-year study on *Thuja occidentalis* clearly illustrated these differences. In horizontal crevices, more seeds and water accumulate on the surface of the soil, which results in a higher rate of seed retention and germination (**Figure 1c**). However, the survival rate of these seedlings is low owing to the limited retention of water in these sites. In contrast, vertical crevices enable hydraulic pressure to channel rainwater down the cliff surface, which results in a greater amount of stable and available water. As a result, the seedlings in vertical crevices survive at higher rates, but the rates of seed retention and germination are lower. Additionally, the seedlings in horizontal crevices are more susceptible to disease and struggle to recover, while in vertical crevices, rockfalls are another cause of seedling mortality (Matthes and Larson, 2006).

## 4 The adaptability of CDPs to cliff habitats

CDPs have evolved distinct characteristics that enhance their growth, development, reproduction, and survival in challenging habitats characterized by steep cliffs, drought conditions, salinity stress, and specialized soil substrates (De Micco and Aronne, 2012; Li et al., 2023). These adaptive mechanisms are essential for maintaining physiological functions and ensuring population persistence in such extreme environments.

### 4.1 The morphology and anatomical characteristics help the CDPs adapt to the unique conditions of cliff habitats

CDPs have evolved sophisticated adaptive strategies at multiple biological levels to survive in harsh cliff environments. At the organismal level, CDPs typically exhibit dwarf morphology or adopt prostrate growth forms (Itoh et al., 2024). Leaf morphological adaptations include reduced size, increased thickness, and waxy cuticles (Radić et al., 2005; Spanò et al., 2013; Pérez-Diz et al., 2023), accompanied by anatomical modifications such as enhanced palisade parenchyma development (Radić et al., 2005), enlarged epidermal cells, specialized water-storing parenchyma, and increased stomatal density (Spanò et al., 2013). Specific adaptations of roots include the development of rhizome structures (Liu et al., 2007) and suberin deposition in root exdermis (Yang et al., 2020). Furthermore, CDPs exhibit stage-specific developmental adaptations, as exemplified by *Primula palinuri* Petagna, which develops specialized hypocotyl hairs during seedling establishment to enhance water absorption capacity (De Micco and Aronne, 2012). These multifaceted adaptations help CDPs adapt to the water- and nutrient-limited environment.

### 4.2 CDPs use a series of physiological mechanisms to adapt to the unique soil and water conditions of cliffs

Recent studies have revealed that approximately 5%-10% of vascular plant species exhibit specific soil preferences (Zhao et al., 2024), a phenomenon particularly evident in certain CDPs with strict edaphic requirements. Among these, *Primulina* species demonstrate remarkable soil specificity, with most taxa being exclusively associated with limestone-derived soils. This adaptation to calcareous substrates is facilitated through specialized physiological mechanisms, particularly in calcium metabolism. These plants have evolved the capacity to store calcium primarily as calcium pectate within their cell walls, a strategy that serves dual protective functions: maintaining membrane stability and preventing cytoplasmic damage through extracellular calcium precipitation (Qi et al., 2013). These unique calcium assimilation and compartmentalization mechanisms likely represent fundamental evolutionary adaptations that enable their survival in specialized soil habitats.

CDPs have evolved diverse strategies to cope with drought stress in cliff environments. A notable example is *Pleurostima purpurea*, which exhibits sophisticated drought avoidance mechanisms. This species initiates stomatal closure at relatively high leaf water content (up to 90%) during moderate drought conditions. This adaptive mechanism establishes a critical temporal buffer that enables rapid restoration of leaf physiological functions upon sudden rehydration of the soil (Aidar et al., 2010). Furthermore, CDPs employ multiple physiological adaptations to mitigate drought stress. These include the upregulation of antioxidant enzyme activities and enhanced biosynthesis of osmoregulatory compounds, which collectively mitigate oxidative damage by scavenging reactive oxygen species (ROS) and maintaining cellular osmotic balance (Yang et al., 2020). Additionally, CDPs regulate their phytohormone profiles in response to drought conditions, particularly through significant accumulation of abscisic acid (ABA), which serves as a key signaling molecule in drought stress responses (Villadangos and Munné-Bosch, 2024).

Plants exhibit remarkable adaptability to cliff habitats through strategic growth adjustments. A prime example is *Sempervivum tectorum*, which demonstrates drought adaptation by minimizing growth and reducing asexual reproduction during dry seasons (Villadangos and Munné-Bosch, 2024), thereby effectively conserving energy. Similarly, *P. palinuri* has developed an adaptive mechanism where its leaves, despite lacking specialized drought-resistant structures, undergo desiccation during summer droughts in their natural habitat (De Micco and Aronne, 2012). Furthermore, CDPs have evolved slow growth strategies to cope with water- and nutrient-deficient soils, this slow growth strategy may also be related to the low N utilization of CDPs (Pérez-Diz et al., 2023). As demonstrated by Zhao et al. (2023a), these plants reduce their growth rate to alleviate environmental stress, showcasing an efficient adaptation to their challenging habitat conditions.

### 4.3 The early growth stages of CDPs is an important phase for their survival

Seedling establishment represents a critical yet understudied phase in the life cycle of CDPs (Aronne et al., 2023). Despite their rarity in natural populations, seedlings play a crucial role in maintaining genetic diversity (Zhao et al., 2023a). The cliff environment poses multiple challenges to seedling survival, including water scarcity, temperature extremes, intense solar radiation, pathogen threats, and spatial limitations. Even when moisture conditions permit seed germination, they often prove inadequate to support extensive seedling growth. While climatic factors significantly influence early seedling development, their impact diminishes with plant maturation (Matthes and Larson, 2006), likely due to the development of stress-tolerant structures. This developmental adaptation is particularly evident in *P. palinuri*, where mature plants develop a suberized root exodermis that prevents water loss. Their rhizomes demonstrate remarkable resilience, capable of continued growth after mechanical damage from environmental forces like strong winds. Additionally, the accumulation of starch in root cells enhances their drought tolerance and other environmental resistances. In contrast, seedlings lack these protective mechanisms, making them particularly vulnerable to environmental stresses compared to mature plants (De Micco and Aronne, 2012).

### 4.4 Sexual and asexual reproduction is crucial for the survival of CDPs

**Figure 2:**
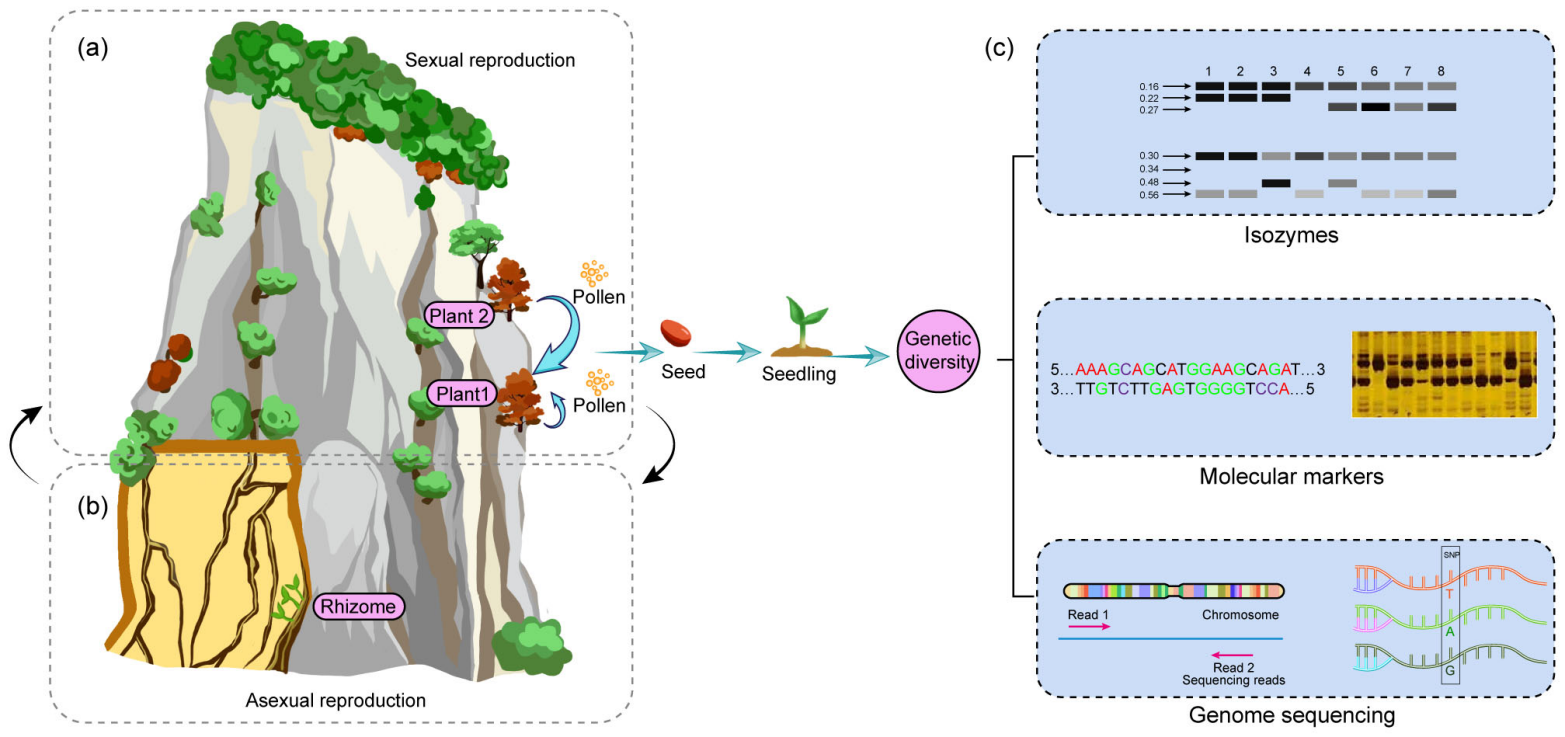
Seeds play a critical role in sustaining the genetic diversity of the population. **(a)** CDPs reproduce sexually through both self-pollination and outcrossing. **(b)** Some CDPs grow in crevices and reproduce asexually via their rhizomes. **(c)** Sexual reproduction is essential to maintain the genetic diversity of the population.

CDPs have evolved remarkable reproductive strategies to ensure species survival in challenging habitats. Sexual reproduction, particularly through pollination, represents a critical yet vulnerable stage in their life cycle (Li et al., 2018), with successful seed production being its ultimate objective. Despite the ecological significance of studying these reproductive adaptations, research is often hindered by the inaccessible nature of cliff habitats. CDPs employ two primary strategies to optimize seed production. First, many species utilize a mixed mating system combining cross- and self-fertilization (**Figure 2a**). This dual approach, observed in species such as *Lonicera oblata* (Wu et al., 2022) and *Opisthopappus* species (Wang et al., 2023), provides both genetic diversity through cross-fertilization and reproductive assurance through self-fertilization (Wang et al., 2023). Furthermore, self-fertilization offers another advantage of increasing homozygosity and facilitating the fixation of adaptive traits such as salt tolerance and late flowering (Itoh et al., 2024). Secondly, CDPs have developed adaptive mechanisms for reproductive organ development. *Taihangia rupestris* exemplifies this adaptation through temperature-dependent differentiation of floral structures. This species produces both hermaphroditic and unisexual flowers, with stamen development at lower temperatures (1-6°C) before April, optimizing pollen production, while pistil development occurs at higher temperatures (6-26°C), ensuring seed maturation by June (Lu et al., 1995, 1996). However, sexual reproduction in CDPs faces significant environmental challenges. Habitat fragmentation, resulting from both natural processes (creating ecological islands) and human activities, disrupts pollen and seed dispersal (Colas et al., 2001; García et al., 2012). Additionally, extreme weather conditions, including temperature fluctuations, intense solar radiation, and strong winds, can adversely affect flowering phenology, pollen viability, and pollinator activity, ultimately impacting pollination success and seed production (Wu et al., 2022).

In addition to sexual reproduction, asexual reproduction is crucial for the survival of CDPs. Many CDPs, such as *T. rupestris* and *Oxyria sinensis*, reproduce asexually through rhizomes, which are particularly important in crevices (**Figure 2b**). These crevices are often interconnected and form a network that promotes the growth and spread of the plants (Lu et al., 1995; Tang et al., 2004; Liu et al., 2007). Rhizome-based reproduction offers several advantages that enhance the plants’ ability to adapt to harsh environments. First, rhizomes enable the formation of extensive clonal colonies, which enables the plants to expand their populations through physiological integration. Secondly, rhizomes help the plants to access more fertile microsites and select the most favorable cliff crevices as they grow. Finally, the interconnectedness of rhizomes can protect the plants from rock fall and erosion, which improves their rates of long-term survival (Liu et al., 2007). In summary, rhizomes are essential for the CDPs to acquire resources, explore suitable microhabitats, expand clonal colonies on cliff surfaces, and contribute to the overall stability of cliff ecosystems.

### 4.5 Molecular technology revealed the mechanism of the adaptation of the CDPs to drought and salt stress

Drought represents a defining characteristic of cliff habitats, and the drought resistance mechanisms in CDPs involve complex physiological processes regulated by multiple genes. Recent studies have identified several key genetic components contributing to drought adaptation. In *Dendrobium catenatum*, the *FAR* (Fatty Acyl-CoA Reductase) genes encode essential enzymes for plant wax biosynthesis, enhancing drought resistance (Ren et al., 2023). Furthermore, transcriptomic analysis of *Opisthopappus taihangensis* roots under drought stress has revealed the upregulation of multiple gene families associated with various protective mechanisms. These findings can be attributed to ABA accumulation, proline and trehalose synthesis, SOD and POD activity, suberin deposition (Yang et al., 2020).

Salt stress constitutes another significant environmental challenge in cliff ecosystems. *Opisthopappus* species have developed sophisticated molecular adaptations, particularly through alternative splicing mechanisms such as skipped exons (SE) and mutually exclusive exons (MXE), to cope with saline conditions. Current research indicates that salt tolerance in these plants is mediated by genes primarily involved in protein phosphorylation, starch and sucrose metabolism, and plant hormone signal transduction (Zhang et al., 2023; Thalmann and Santelia, 2017; Han et al., 2024).

### 4.6 Genetic diversity is essential for the survival and conservation of CDPs

Genetic diversity is crucial for species survival and environmental adaptation (Ding et al., 2015; George et al., 2009). High genetic diversity enhances evolutionary potential, while low diversity limits adaptability to environmental changes (Binks et al., 2015). For example, some CDP species, like *C. acerifolia* (López-Pujol et al., 2005; Zhao et al., 2023a), *Asperula naufraga* (Valli et al., 2021), and *Antirrhinum subaeticum* (Jiménez et al., 2002), exhibit low genetic diversity, while others, such as *O. taihangensis* (Guo et al., 2013) and *T. rupestris* (Tang et al., 2010; Duan, 2013; Wang et al., 2011; Li et al., 2018), show higher diversity. Moreover, genetic diversity is influenced by factors such as cliff crevice variability (such as differences in size, depth, and microclimatic conditions), environmental pressures, seedling growth, pollen and seed dispersal, and reproductive biology (George et al., 2009; Neri et al., 2021; Zhao et al., 2023b). In China’s Taihang Mountains, *C. acerifolia*, *T. rupestris*, and *O. taihangensis* coexist but display varying genetic diversity levels. *C. acerifolia’s* low diversity may result from limited seed dispersal and few wild seedlings (Zhao et al., 2023), whereas *T. rupestris’s* high diversity is likely due to its ancient origins and mixed reproductive strategies (Wang et al., 2011). Similarly, *O. taihangensis* benefits from wind and gravity-aided seed dispersal, enhancing its genetic diversity (Guo et al., 2013). Evaluating genetic diversity is essential for CDP conservation and management. However, current methods, such as isozymes (López-Pujol et al., 2005), ISSR (Guo et al., 2013), and simplified genome-derived SSRs (Zhao et al., 2023a), may not fully capture genetic diversity due to insufficient genomic data (**Figure 2c**). To overcome this limitation, future research should focus on generating comprehensive genomic data for CDPs, which will allow for a more accurate assessment of their genetic diversity.

Genomic data have significantly advanced our understanding of the population and evolutionary genetics of plants. The genomes of CDPs provide critical insights into their adaptive mechanisms and serve as essential resources for genetic diversity research. Plant genomes include the nuclear, chloroplast, and mitochondrial genomes, and to date, eight CDP species have been sequenced (**Table 1**). Chromosome-level genome sequencing is particularly valuable for accurately assessing genetic diversity, elucidating adaptation mechanisms, and guiding conservation strategies (Li W. G. et al., 2024). For instance, the genome sizes of *Taihangia rupestris* var. *ciliata* from the Taihang Mountains and *Scaevola tacada* from seaside cliffs are 769.5 Mb and 1.1 Gb, respectively (Li W. G. et al., 2024; Li et al., 2023). Both species exhibit a high abundance of long terminal repeat retrotransposons (LTR-RTs), suggesting these elements play a key role in their adaptation to extreme environments. *T. rupestris* var. *ciliata*, adapted to mountain cliffs, faces drought and cold stress. Its resilience is likely mediated by transposable elements (TEs), LTR-RTs, and oxidative phosphorylation genes (Li W. G. et al., 2024). In contrast, *S. tacada*, inhabiting seaside cliffs, endures intense light and salt stress, with the *FAR1* gene (FAR-RED-IMPAIRED RESPONSE1) playing a crucial role in its coastal adaptation (Li et al., 2023). These findings highlight the importance of genomic studies in understanding CDP adaptation and informing conservation efforts.

**Table 1:** CDPs with reported genomic information.

Species	Plant type	Genome type	Genome size	Distribution	Environment	References
*Petrocosmea* *qinlingensis*	Herb	Chloroplast genome	153865 bp	Qinling Mountains, China	Shady limestone cliff	Li C. Q. et al., 2024
*Scaevola taccada*	Shrub or small tree	Chromosomal-scale genome	1.11 Gb	Widespread through the Pacific and Indian Oceans	Costal cliff	Li et al., 2023
*Taihangia rupestris* var. *ciliata*	Herb	Chromosomal-scale genome	769.5 MB	Taihang Mountains, China	Limestone cliff	Li W. G. et al., 2024
*T. rupestris* and *T. rupestris* var. *ciliata*	Herb	Chloroplast genome	155558 bp and 155479 bp	Taihang Mountains, China	Limestone cliff	Feng et al., 2022
*Euphorbia hainanensis*	Bush	Chloroplast genome	163977 bp	Hainan, China	Clifftop	Zhang et al., 2019
*Clematis acerifolia*	Shrub	Mitochondrial genome	698247 bp	Taihang Mountains, China	Shady limestone cliff	Liu D. et al., 2023
		Chloroplast genome	159552bp			Xiang et al., 2019
*Opisthopappus taihangensis* and *O. Longilobus*	Herb	Chloroplast genome	151117 bp and 151123 bp	Taihang Mountains, China	Limestone cliff	Zhang et al, 2022

## 5 Monitoring and phenotypic studies of CDPs

Accessing CDPs is extremely challenging owing to the steep and rugged nature of the cliffs where they are found. As a result, a variety of auxiliary methods and equipment are necessary to gather sample and reliable data from locations that are otherwise inaccessible to researchers. These auxiliary survey methods have evolved through three main phases, including telescope-based observation, rock climbing, and the use of drones equipped with imaging tools (**Table 2**). Telescope observations enable the remote monitoring of CDPs, but the limited field of view and inability to study large areas make this method unsuitable for extensive research (Alfaro-Saiz et al., 2019). Rock climbers, while capable of observing, photographing, and sampling plants during their ascent, face significant risks and must possess advanced climbing skills to perform these tasks safely (Boggess et al., 2021; March-Salas et al., 2023). Fortunately, drones have emerged as a highly effective tool to study CDPs. Equipped with imaging devices and robotic arms, drones can conduct aerial photography and material sampling over large areas; this addresses the limitations of telescopic and rock-climbing methods (Zhou et al., 2021; La Vigne et al., 2022). One particularly valuable technique is Nap-of-the-Object Photography, which enables drones to capture high-resolution images with precision, record accurate spatial positioning, and facilitate the efficient collection of data. For instance, Nap-of-the-Object Photography was used to study the habitat and growth of *C. acerifolia*. The results revealed that individual plants exhibit similar morphological characteristics, with most located on cliffs facing the west or northwest and growing in semi-shady environments. Additionally, several cliff crevice parameters, including altitude, height, orientation, width, and angle, were identified, thus, providing insights into the most suitable conditions for *in situ* conservation and guiding recommendations for cultivation practices, such as sowing and transplanting *C. acerifolia* (Gao et al., 2024). Overall, the integration of these advanced technologies has significantly advanced our investigation of CDPs, particularly those growing in remote, high-altitude, and steep environments. These innovations have not only deepened our knowledge of the plants’ biology but also provided critical data that supports their conservation and management.

**Table 2:** The advantages and disadvantages of three observation methods for CDPs.

Observation Method	Advantages	Disadvantages	References
Telescope	Remote observation; Do not interfere with plants	Limited view and sampling	Alfaro-Saiz et al., 2019
Rock climbing	Close observation and sampling	Dangerous; Skilled technique required; May disturb cliff plants	Boggess et al., 2021; March-Salas et al., 2023
Drone technology	Close observation and sampling in a large range	Operational technology required	Gao et al., 2024

Furthermore, long-term monitoring is essential for understanding the ecological dynamics of CDPs. An 18-year long-term study of *T. occidentalis* populations on cliff faces demonstrated that while weather conditions predominantly influenced seed germination and seedling establishment stages, their impact significantly decreased following successful establishment (Matthes and Larson, 2006). Given the characteristic slow growth rates (Pérez-Diz et al., 2023), prolonged life cycles (Zhao et al., 2023a), and extreme habitat adaptations (Itoh et al., 2024) of CDPs, short-term investigations may yield misleading or contradictory findings about these specialized plants. Despite this critical need, long-term monitoring studies of CDPs remain notably scarce in contemporary scientific literature, representing a significant gap in our understanding of these unique ecological systems.

## 6 The conservations of the CDPs

### 6.1 Maintaining genetic diversity is essential for CDPs

**Figure 3:**
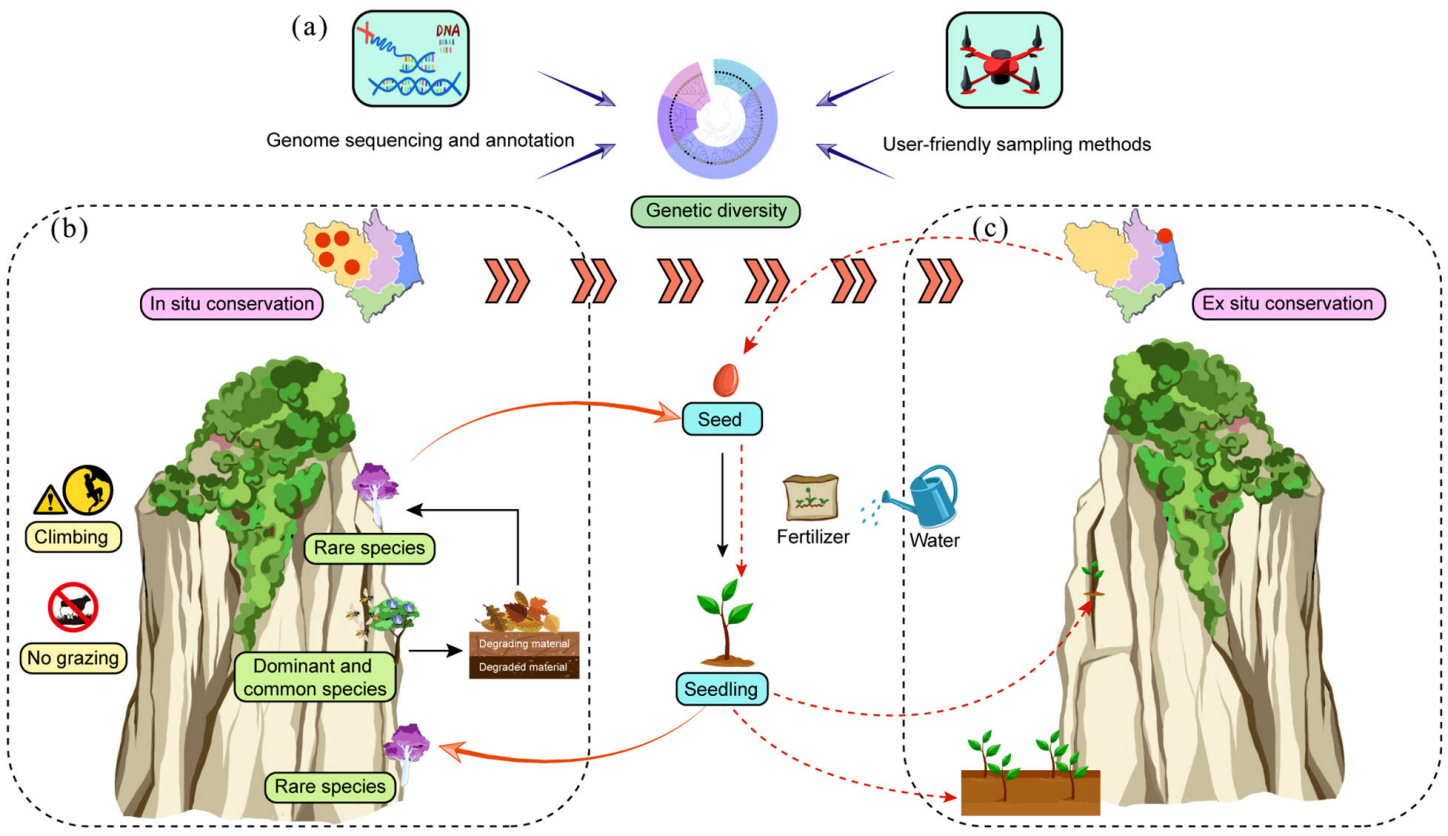
Evaluating genetic diversity is crucial for the conservation of CDPs. **(a)** Genome sequencing provides a reliable reference for genetic studies. **(b)** *In situ* conservation of CDPs involves protecting both their habitats and the entire cliff ecosystem. **(c)** *Ex situ* conservation of CDPs should focus on the role of seeds and seedlings. Owing to the unique characteristics of CDPs, artificial seed sowing requires the careful consideration of water and nutrient availability.

The survival of CDPs is increasingly threatened by their highly specialized habitats, which are often fragmented and unstable (Clark-Tapia et al., 2021). Genetic diversity is critical for their long-term survival, serving as a key indicator of adaptability and evolutionary potential (Ding et al., 2015). Current methods for assessing genetic diversity, such as isozymes (López-Pujol et al., 2005) and molecular markers like SSRs (Zhao et al., 2023a) and SNPs (Ye et al., 2021), often rely on limited markers, potentially underestimating genetic variation. Furthermore, reference genomes for most CDPs remain unavailable (**Table 1**), hindering comprehensive genetic analyses. The development of high-quality, *de novo* assembled genomes would provide robust genomic data, improving the accuracy of genetic diversity assessments and population structure analyses (**Figure 3a**). Such resources are expected to become more accessible with the decrease of sequencing cost, enabling deeper insights into CDP genetics. Sampling representativeness is another critical factor in genetic diversity studies. However, accessing CDPs for sampling is often risky and impractical (Zhou et al., 2021), and care must be taken to avoid collecting clones from the same crevice (Tang, 2004; Tang et al., 2004; Zhao et al., 2023a). Recent advancements in unmanned technologies have facilitated safer and more efficient observation and sampling of CDPs (Gao et al., 2024). Moving forward, user-friendly sampling methods and interdisciplinary collaboration will be essential for large-scale, comprehensive genetic diversity and taxonomic studies (**Figure 3a**). These efforts will enhance our understanding of CDP adaptation and inform effective conservation strategies.

### 6.2 Developing a range of *in situ* and *ex situ* conservation measures for CDPs Are necessary

In practical conservation efforts, it is essential to comprehensively consider the factors influencing the survival of CDPs (**Figure 3b**). Conservation strategies should not only focus on rare species but also encompass common CDPs, as they play critical roles in maintaining ecosystem stability and supporting biodiversity. These common species reduce rock erosion, support species colonization, and contribute to nutrient cycling through biomass decomposition, enriching the cliff ecosystem. Additionally, their abundance attracts pollinators, enhancing the reproductive success of both rare and common plants (March-Salas et al., 2023). Therefore, conservation strategies should adopt a holistic approach, prioritizing the preservation of the entire cliff ecosystem, including both rare and common species. It is noteworthy that rock climbing, a globally popular recreational activity, has the potential to disrupt cliff ecosystems, although studies investigating its impact on CDPs have produced inconsistent findings. Some studies indicate that climbing reduces vegetation cover, species richness, and alters ecosystem composition (Lorite et al., 2017; de Castro-Arrazola et al., 2021), while others find no significant effects (Boggess et al., 2017, 2021). These discrepancies may stem from varying climbing intensities (Boggess et al., 2017) or natural differences in cliff vegetation (Boggess et al., 2021). As a result, balancing recreational rock climbing with the preservation of cliff ecosystem integrity is very important in CDP conservation.

*Ex situ* conservation can be effectively achieved through the reintroduction of artificially propagated materials, such as seeds or seedlings, into native habitats or by establishing protected areas like botanical gardens. Usually, mature plants are often unsuitable for reintroduction due to their deep roots in unstable cliff crevices. Seeds, in particular, are ideal for propagation and protection, as they suffer less damage and are easier to handle (Liu D. Y. et al., 2023; Zhao et al., 2023b). The survival rate of reintroduced materials depends significantly on the specific location of cliff crevices chosen (Gao et al., 2024). A successful example is the reintroduction of *Hubbardia heptaneuron* in 2009, where seeds were used, and damp, dark areas near waterfalls with dense vegetation were identified as optimal conditions (Duvall et al., 2017). Furthermore, water management is crucial during artificial propagation, as CDPs typically thrive in arid environments (Li W. G. et al., 2024). Additionally, nutrient acquisition in CDPs may rely on fungi, which have substrate preferences, suggesting that nutrient availability influences their growth (Toghueo et al., 2023; Algora Gallardo et al., 2021). In summary, effective management of propagation materials and tailored substrate conditions are essential for the successful conservation of CDPs.

### 6.3 Adaptation and conservation strategies for cliff-dwelling plants in the face of global climate change: evolution, migration, and genetic resource protection

The drastic impacts of global climate change, including increased droughts, storms, and extreme temperatures (Lindenmayer and Laurance, 2016; Shinozaki and Yamaguchi-Shinozaki, 2024), are reshaping ecosystems worldwide. In response to these significant environmental changes, plants typically migrate to more suitable areas or undergo self-evolution to adapt to new conditions (You et al., 2024). On the one hand, CDPs have evolved into various ecological forms to survive harsh environments (Itoh et al., 2024) or variants (Yan et al., 2016). As climate change progresses, it is anticipated that CDPs will continue to evolve into even more diverse forms or potentially new species adapt to increasingly extreme conditions. Alternatively, habitat fragmentation owing to climate change, coupled with the specific habitat requirements, may hinder the ability of CDPs to naturally migrate through seed dispersal or asexual reproduction (Clark-Tapia et al., 2021). In such cases, artificial propagation techniques can assist in relocating the CDPs to more suitable environments. For instance, to address extreme drought conditions, artificial water supply systems could be utilized. The effects of rising temperatures could be mitigated by relocating the CDPs from low-latitude areas to cooler, higher-latitude regions. Ultimately, given the severity of global climate change, protecting the genetic resources of CDPs is of the utmost importance. Establishing specialized germplasm banks for these plants is a crucial strategy for safeguarding this unique and habitat-specific plant group.

## 7 Research limitations and prospect

### 7.1 Critical research limitations in understanding CDPs: habitat monitoring, physiological adaptations, and molecular mechanisms

Research on CDPs primarily focuses on their adaptation mechanisms to cliff environments and conservation efforts. However, due to the inaccessibility of cliff habitats and the slow growth rates of CDPs, observation and sampling remain challenging, leaving several areas understudied. First, Conducting long-term monitoring is an essential approach for studying cliff ecosystems, understanding plant-environment interactions, and conserving plant species. (1) Ecosystem networks: mapping plant-animal-microbe-habitat interactions; (2) Survival mechanisms: high-resolution phenotyping of stress responses to chronic/acute environmental stresses. (3) Conservation efforts: tracking habitat dynamics, soil-water parameters, and population viability under different conditions. Furthermore, effective implementation requires renewable-powered equipment and automated data workflows; Second, physiological mechanisms including nutrient utilization in depleted environments, water absorption and efficiency, overwintering strategies, root-microbe interactions in cliff crevices, photosynthetic characteristics, and shade tolerance are scarce. Third, molecular mechanisms, especially resistance-related genes, as well as lifecycle bottlenecks, are significantly lacking in most CDPs (Aronne et al., 2023).

### 7.2 Future directions in CDP research: innovative tools and interdisciplinary methodologies

The development of innovative technologies and tools presents promising solutions to current research limitations in CDP studies, serving as a pivotal direction for future investigations. These advancements should address cliff habitat challenges, such as energy-efficient real-time image acquisition systems for monitoring microhabitat dynamics during sustainable ecological observation; standardized laboratory cultivation protocols to obtain experimental materials for physiological studies; and molecular genetic transformation platforms enabling cellular-level exploration of stress adaptation mechanisms. Each technological pathway specifically counteracts the inaccessibility of cliff ecosystems and the slow growth constraints inherent to CDPs.

Interdisciplinary integration is also essential for studying CDPs and their unique habitats. Long-term monitoring demands technologies spanning microecology, engineering mechanics, drone systems, and AI-driven data analytics to automate environmental sensing in inaccessible terrains. Meanwhile, physiological investigations require convergence of ecology, soil science, and phenomics to decode nutrient dynamics and stress adaptation traits. At the molecular level, single-cell omics, epigenetics, and bioinformatics jointly map vertical-microclimate-induced gene regulation and stress signaling networks.

## 8 Conclusion

This review presents a comprehensive overview of CDPs, covering their ecological significance, potential applications, environmental influences, plant adaptation mechanisms, observation methods, and conservation strategies. Looking ahead, the review emphasizes two crucial directions for future research: the development of advanced tools and methodologies specifically tailored for studying CDPs, and the application of interdisciplinary approaches to deepen our understanding of the habitat adaptation mechanisms of plants and to strengthen conservation efforts. This integrated perspective highlights the necessity for interdisciplinary research across various scientific fields to address the unique challenges posed by cliff ecosystems.
